# Patient-Identified Solutions to Primary Care Access Barriers in Canada: The Viewpoints of Nepalese Immigrant Community Members

**DOI:** 10.1177/21501319221141797

**Published:** 2022-12-08

**Authors:** Rudra Dahal, Jessica Naidu, Bishnu Bahadur Bajgain, Kalpana Thapa Bajgain, Kamala Adhikari, Nashit Chowdhury, Tanvir C. Turin

**Affiliations:** 1 Nepalese-Canadian Community, Calgary, AB, Canada; 2 University of Lethbridge, Lethbridge, AB, Canada; 3 University of Calgary, Calgary, AB, Canada; 4 Alberta Health Services, Calgary, AB, Canada

**Keywords:** immigrants, Nepalese, primary care, solutions, healthcare access, health inequalities, community-engaged research

## Abstract

**Background:**

Accessing healthcare for immigrants in Canada is complicated by many difficulties. With the continued and upward trend of immigration to Canada, it is crucial to identify the solutions to the barriers from the perspectives of different immigrant communities as they encounter them including the relatively smaller and less studied population groups of immigrants. As such, Nepalese immigrants in Canada are a South Asian ethnic group who have their own distinct language, culture, and socio-economic backgrounds, however, their experience with accessing healthcare in Canada is scarce in the literature.

**Methods:**

We conducted 12 focus group discussions with first-generation Nepalese immigrants who had experiences with primary care use in Canada. Informed consent and demographic information were obtained before each focus group discussion. The verbatim transcription of the focus groups was analyzed using thematic analysis.

**Results:**

The participants expressed a range of potential solutions to overcome the barriers, which we presented using the socio-ecological framework into 4 different levels. This includes individual-, community-, service provider-, and government/policy-levels. Individual-level actions included improving self-awareness and knowledge of health in general and navigating the healthcare system and proactively improving the language skills and assimilating into the Canadian culture. Examples of community-level actions included community events to share health information with immigrants, health literacy programs, and driving/carpooling to clinics or hospitals. Actions at the service provider level were mainly focused on enhancing communications, cultural competency training for providers, and ensuring to hire primary care workforce representing various ethnocultural backgrounds. Overall, focus group participants believed that the provincial and federal government, as appropriate, should increase support for dental and vision care support and take actions to increase the healthcare capacity, particularly by employing internationally graduated health professionals.

**Conclusions:**

Access to primary care is essential for the health of immigrant populations in Canada. Individuals, community organizations, health service providers, and governments need to work both individually and collaboratively to improve immigrants’ primary care access.

## Introduction

Canada has a universal and publicly funded healthcare system that aims to provide equitable access to health services, including primary care to all Canadians regardless of their immigration status or ability to pay.^1^ The provinces and territories administer most of Canada’s healthcare services and are expected to meet national principles set out under the Canada Health Act.^1^ Each provincial and territorial health insurance plan covers medically necessary physician and hospital services that are provided without direct charges at the point of service. The provincial and territorial governments fund these services with assistance from federal funding through cash and tax transfers.^2,3^ However, within the publicly funded healthcare system, health expenditures and delivery systems vary across the provinces and territories. This is, in part, due to the differences in the services that each province and territory covers.^2,3^

Primary care is the first point of contact with healthcare for any individual including immigrants. Services include preventative services, treatment of common diseases and injuries, basic emergency services, referrals to and coordination with other levels of healthcare, pediatric care, primary maternity care, and rehabilitative services.^4^ Access to healthcare implies timely use of healthcare services to achieve the best health outcomes.^5^ Despite the universal healthcare system that is provincially administered in Canada, immigrant populations access healthcare services less, compared with Canadian-born populations.^6,7^ Immigrants generally have better health than their Canadian-born counterparts upon their arrival, a phenomenon known as the “healthy immigrant effect.”^8^ However, their general health deteriorates after a few years of migration.^9-11^ Research in this area suggests that immigrant populations have unmet health needs due to barriers they face while accessing primary care.^12,13^ Examples of such barriers are language difficulties, cultural differences, lack of knowledge of navigating the healthcare system in the host country, gender expectations, geographic location, and out-of-pocket costs.^6,8^ Several previous studies have identified barriers faced by immigrants while accessing primary care in Canada;^12,14,15^ however, there is not much research on identifying solutions to these barriers.^16,17^ There is also a gap in research identifying the key individuals and organizational entities (from individual to community to policymaker levels) and the ways of interaction between them that may be required to implement the solutions successfully.^17^

There is a paucity of community-engaged research focusing on immigrant communities, and barriers and solutions to primary care access.^18^ This type of research is valuable because it ensures that the assessment, planning, implementation, and evaluation of community health issues and solutions prioritize the expressed needs of the communities.^19,20^ However, such research needs to involve specific communities in the research to effectively identify and develop solutions that work for them.^21,22^ Engaging and involving community members in health research ensures that community members’ voices will be reflected in the said research.^19,23,24^ This further ensures that research-based decision-making will reflect the voices of the community members and is more likely to meet their health needs.^25,26^ Such an approach is crucial for a multicultural nation like Canada, where one general solution may not work for all diverse communities.^21,27^

We focused in this study on a very specific and rarely studied immigrant community—the Nepalese immigrants in Canada. The number of Nepalese populations immigrating to Canada has been accelerating. According to Statistics Canada 2016, there were 9870 Nepalese Canadians who resided in Canada in 2011, and the number reached 14 390 in 2016, among them 5175 residing in the province of Alberta.^28^ Like many other racialized, newcomer communities, members of the Nepalese community are often not born, raised, and educated in Canada, and speak English as a second language. Thus, they are often at risk of experiencing marginalization, particularly when accessing healthcare.^29^ While the Nepalese community somewhat differs from other South Asian communities in terms of culture, religion, food habits, and approaches to health, Nepalese and other South Asian communities in existing literature are often viewed through a lens of homogeneity.^30,31^ This may lead the stakeholders to develop solution measures with the postulation of one size fits all, which is not an appropriate approach. Therefore, this study aims to capture the perspectives of Nepalese immigrants in Calgary, Alberta on improving access to primary care for their community, specifically individual responsibilities, community responsibilities, service providers’ responsibilities, and policy-level responsibilities in creating solutions to barriers they have identified. While our aim is not to generalize our findings to the Nepalese communities across Canada, our findings may be transferable to the Nepalese and other similar communities in Calgary.

## Methods

### Framework—Socio-Ecological Model

The Socio-ecological model (SEM) is a useful framework for the study of community health.^32^ The SEM considers how a range of behavioral, social, and environmental factors from individual, community, governmental, and broader societal contexts, impact health. Furthermore, the SEM assesses the interplay between these factors and the resultant health outcomes.^32^ The SEM recognizes that health is shaped by context, health and health behaviors are a result of more than just individual behavior, and that there are many layers that act upon individuals, shaping their behaviors and resulting health.^32^ Thus, it is a useful framework for understanding the factors that mitigate and facilitate health within a community, in this case, the Nepalese immigrant community in Calgary, Canada. Applying the SEM to our study, we organized the proposed solutions into individual responsibilities, community-level responsibilities, service provider-level responsibilities, and policy/government-level responsibilities to mitigate barriers to access to primary care.

### Outreach and Data Collection

We undertook a community-engaged approach for this study using focus group discussions (FGD) as the data collection tool.^33,34^ Ethical approval for this study was obtained from the Conjoint Health Research Ethics Board at the University of Calgary [REB15-2325]. The principal investigator and team members have been building a program of community engaged research focusing on equitable access to primary care for immigrant/racialized communities in Canada. A small group of members of the Nepalese-Canadian community, who took the role of community scholar and citizen researcher, co-developed this study including the study design, development of the focus group questionnaire, analysis, interpretation of the findings, and writing manuscripts with the principal investigator’s program of research. These Nepalese-Canadian community scholars and citizen researchers have had a history of volunteering within their community through different activities. They led various rounds of conversations with the Nepalese community about healthcare access difficulties with community members. Their familiarity with the community and its dynamics situated them as insider researchers. In concert, the principal investigator and other team members were situated as trusted and accepted outside champions or outsider researchers who actively facilitate the participatory process, enable capacity building training, and mobilize resources for research with the community.

Our team used a community-engaged approach to understand the effects of individuals’ attitudes, beliefs, and behavior on health within their social, cultural, and environmental contexts.^35^ Furthermore, this approach allows for translating scientific discovery into critical practice and public health initiatives. It creates a new way of generating knowledge to improve health outcomes in the community. Also, it is a unique method of scientific discovery that brings transdisciplinary teams together which study the community’s health problems in a real-world setting.^35^ We engaged with the Nepalese-Canadian community champions including the leadership of the Nepalese Community Society of Calgary (NCSC) and community members for this study. We informed and discussed with them the study purpose and objectives and with their support, we disseminated our recruitment materials to the adult Nepalese immigrants in Calgary. Once we had contact with the potential participants, we explained to them the research purpose and their roles. If they chose to participate, a suitable time and place were selected that worked for each participant of a focus group. The first author of this manuscript facilitated the FGD sessions and the second and third authors acted as note-takers. Written informed consent was obtained from each participant and basic socio-demographic information was collected before proceeding with the FGD.

To capture the perspectives of the participants, we asked them about any proposed solutions which they think will mitigate the challenges they faced while accessing primary care. During the FGDs, to set the context initially we started the discussion by asking about their general experience in accessing primary care in Canada and the challenges they faced. Then we asked about the participants’ views on solutions. To make it easy, the facilitator picked barriers that were already mentioned by them and asked the participants what their thought was to overcome these barriers in terms of individual responsibility, community responsibility, service provider’s responsibility, and policy-level responsibilities.

FGDs were conducted between February and June 2019. Twelve FGDs (6 male and 6 female groups) were conducted with a total number of 68 participants. Each group consisted of 5 to 7 participants. A trained bilingual and bicultural facilitator conducted the FGDs. All FGDs were audio-recorded, and the facilitator took field notes as well. Each FGD lasted for about 1.0 to 1.5 h. Audio recordings were first transcribed and then translated into English (if participants expressed their opinion in Nepalese).

### Data Analysis

We conducted a deductive thematic analysis^36^ of the transcripts to illustrate potential solutions recommended by the Nepalese immigrants we spoke with. We used Braun and Clarke’s^36^ approach to thematic analysis that entails 6-steps; (a) familiarizing with the data; (b) generating preliminary codes; (c) searching for themes; (d) reviewing the themes; (e) naming and defining themes; and (f) producing the report.^36^

*(a) Familiarizing with the data*: We transcribed the recorded data verbatim and compared them with recorded audio and field notes taken during each FGD. The transcription and analysis were done manually without using any instruments. The transcripts were discussed several times among the research team for accuracy as well as compared with the field notes. The transcripts were examined to see if captured the discussions appropriately to answer our research questions.
*(b) Generating preliminary codes*: The facilitator of the first phase of FGDs (3 male groups and 3 female groups) coded the relevant and meaningful excerpts from the data and discussed it with the Principal Investigator (PI) to ensure that the coding process is being done properly. The remaining 6 FGD recordings were also transcribed and coded by the same facilitator and consulted with the PI to resolve any discrepancies.
*(c) Searching for themes*: After the completion of coding, initially, we populated a large table with all our results. We then narrowed it to related information only and resized the table. At this level, we looked for appearing patterns within the codes and we identified them as preliminary themes. As we were using the SEM framework, our identified themes started to correspond with the different tiers of this framework.^37^ This model helped us understand the determinants of health-related behavior, often organized into the following tiers: individual, community, systems, and broader socio-political context.^32^ The model can be adapted to fit the needs of various community health inquiries. For our purposes, we examined the individual, community, service provider, and government tiers. After the initial analysis of the codes, our research team gathered relevant themes and detailed them for further analysis.
*(d) Reviewing the themes*: Our research team created a thematic map for examining and analyzing our FGD data. The themes were discussed by the different members of the research team and scrutinized from different perspectives. We conducted member checking during the phase of theme finalization with 10 community members who participated in the FGDs. We intended to ensure the credibility of the codes and themes and relevance to their perspectives. These discussions also contributed to the naming and defining of the themes for the final reporting.
*(e) Naming and defining themes*: Here, we finalized the details of themes and attached names to them followed by a discussion within the research team, reflecting our research objectives.
*(f) Producing the report*: We reported the identified themes and supported that with relevant quotes from our participants. Subsequently, we discussed the results in light of previous literature and the SEM framework to understand the findings and the implications in practice.

## Results

### Demographic Details

As presented in Table 1, the participants [n = 68 (Male = 34; Female = 34)] were first-generation Nepalese immigrants who had been residing in Calgary for 5 to 9 years at the time of the study. All of them spoke the Nepalese language at home and were competent in English as the second language. All Families typically consisted of 4-a husband, a wife, and 2 children. The majority of participants attained graduate-level education. Most of them were employed full-time. All of them had family doctors and extended health insurance coverage.

**Table 1.** Demographic Characteristics of Participants (n = 68).

Characteristics	Male	Female
Age group		
≤25 years	0 (0%)	7 (21%)
26-35 years	7 (21%)	10 (29%)
36-45 years	18 (53%)	13 (38%)
46-55 years	5 (15%)	4 (12%)
56-65 years	2 (6%)	0 (0%)
≥66 years	2 (6%)	0 (0%)
Marital status		
Married	34 (100%)	25 (74%)
Single/separated/divorced	0 (0%)	9 (26%)
Total family members		
1 member	0 (0%)	3 (9%)
2 members	3 (9%)	9 (26%)
3 members	9 (47%)	2 (6%)
4 members	20 (59%)	18 (53%)
≥5 members	2 (6%)	2 (6%)
Number of dependents in family		
No dependents	5 (15%)	12 (35%)
1 dependent	4 (12%)	8 (24%)
2 dependents	20 (59%)	12 (35%)
3 dependents	3 (9%)	2 (6%)
≥4 dependents	2 (6%)	0 (0%)
Level of education		
Graduate level	24 (71%)	15 (44%)
Bachelor level	8 (24%)	9 (26%)
Some college levels	2 (6%)	8 (24%)
School levels	0 (0%)	2 (6%)
Employment status		
Full-time job	28 (82%)	20 (59%)
Part-time job	2 (6%)	5 (15%)
Self-employed	2 (6%)	0 (0%)
Student	2 (6%)	5 (15$)
Looking for job	0.(0%)	4 (12%)
Length of stay in Canada		
≤5 years	7 (21%)	10 (29%)
5-9 years	16 (47%)	17 (50%0
10-14 years	6 (18%)	6 (18%)
≤15 years	5 (15%)	1 (3%)
Yearly household income		
≤$25 000	2 (6%)	7 (21%)
$26 000-50 000	8 (24%)	10 (29%)
$51 000-75 000	12 (35%)	6 (18%)
$76 000-95 000	6 (18%)	9 (26%)
≥$96 000	6 (18%)	2 (6%)

## Proposed Recommendations

Participants proposed a variety of recommendations to mitigate the barriers to accessing primary care. We used the SEM model to interpret our discussion findings, thus, we have organized our results as per the SEM as well as during the thematic analysis, the recommendations were organized using the SEM model as follows:

1. Individual-level responsibilities
2. Community-level responsibilities
3. Service provider-level responsibilities
4. Policy/government-level responsibilities

## Individual-Level Responsibilities

Individual-level responsibilities are those actions that an individual can take to improve their primary care access. According to our participants, community members can take some personal initiatives to lessen barriers to accessing primary care. First of all, our participants encouraged new immigrants to improve their English language proficiency, which helps to improve the communication barrier. FGD participants recommended enrolling in English classes provided by various settlement-related organizations. Then, they discussed self-education, which could be achieved by reading various healthcare-related brochures, pamphlets, magazines, and books. Also, people can learn about the healthcare system and its navigation in their province/territories by watching YouTube videos, by searching on Google, talking to their friends, and participating in group talks. When individuals gain knowledge it helps them to overcome hesitation. The emphasis here is on reducing perceived barriers through education efforts and improving the individual perception of accessing healthcare.

*“Self-educate on personal health and healthcare needs. I think everybody has the right to know about their health, so all should self-educate themselves by asking questions to doctors, reading health-related papers, watching YouTube videos, and searching on Google.” [FGD # 3, Participant # 1].*

Then, our participants discussed adjusting to a new culture. FGD participants urged that a person should adapt accordingly while there are no other options even though adapting to a with new culture is not easy. It takes time to realize the new situation and environment.

*“It was very hard for me to adopt a completely different culture here. I was shocked to see the Canadian culture. But I did not have a choice. So, I started learning English by enrolling immigrant society of Calgary, then started to talk to classmates and teachers to improve my speaking level. It took a while to adapt to this change, but I did it, so be patient! And continue to learn new things, and you will succeed. In my experience, our attitudes and beliefs, level of knowledge, and determination play a vital role to adapt a new environment” [FGD # 12, participant # 6].*

Therefore, individual-level responsibilities prepare a person to adapt to a new situation by expanding their level of knowledge and preparing them to realize the new culture and changing environment which is vital to accessing primary care. In these ways, personal responsibilities assist individuals to mitigate barriers to accessing primary care.

## Community-Level Responsibilities

The participants pointed out the importance of community-level organizations’ involvement in improving primary care access. As community-level entities in the cities/provinces/territories, for instance, the Nepalese Community Society of Calgary (NCSC) and similar culture/faith-based organizations may have certain capacities to facilitate primary care access for the members of their communities. For example, community organizations could facilitate health information-sharing sessions for community members on providing an overview of the healthcare system in their provinces/territories, acquaint them with various health professionals from their communities, and the culture and beliefs around health and wellbeing in the localities and Canada, in general. Most of the participants mentioned the importance of community organizations in helping newcomers by providing important health-related information for settlement in the early days of migration to Canada. Participants pointed out that community organizations can facilitate community events, allowing newly arrived immigrants to connect, share their feelings about their new environment, and share tips to ease the primary care access process. Participants suggested that community organizations could provide health literacy and awareness programs to answer important common questions about how one can better access or navigate the local health system.

*“Community organizations can organize health promotion programs such as how to be healthy. What are existing health services? How can we access health services? Additionally, the community can collaborate with other organizations and add different programs such as smoking cessation programs, and healthy eating practices. They can coordinate with AHS and can run mental health and counseling programs using volunteers from the Nepalese community” [FGD # 7, participant # 2].*

In addition to information sharing, these programs could provide hands-on guidance such as helping to find a family physician and carpooling to the appointments. Also, during FGD it was tremendously discussed to provide translator services for people with limited English language proficiency. Some of our participants suggested creating an online forum where people can ask questions and get an answer to healthcare-related inquiries. Overall, several participants stressed that community organizations could play a vital role in easing challenges to accessing primary care by facilitating various programs for newcomers.

*“In my experience, community organizations (NCSC) can organize health awareness and information sessions. For instance, many immigrant communities have community groups that can provide information sessions and health awareness programs, which can assist newcomers to know the basic system and how to utilize the healthcare system here. To do so community organizations such as NCSC can distribute brochures and leaflets in their native Nepali language which is a good resource to get information to know the system” [FGD # 8, participant # 1]*

## Service Provider-Level Responsibilities

Several FGD participants strongly believed that primary care service providers such as physicians, nurses, and other allied healthcare professionals can substantially improve access to primary care for immigrant populations. Many participants discussed the importance of communication between physicians and patients in healthcare settings to ensure the best health outcomes for patients. Given the language barriers between immigrants and service providers in healthcare settings, participants felt that doctors fail to effectively hear immigrants’ complaints at visits and checkups. A recommended solution was in-house translators at service provider locations to overcome these language barriers and resulting communication gaps. Alternatively, physicians’ offices could coordinate translation services when they are aware of language barriers.

*“I think doctors and hospitals need to have an interpreter to serve a better multicultural/diverse population. Here is the problem not all people can speak English and are unable to understand what the medical professionals are talking about in the office. If there is a misunderstanding among them, the treatment plan may alter and may be dangerous. In this case, the service provider can arrange a translator. For this, they can ask patients during appointment booking if they need an interpreter and can ask patients self to bring them or arrange it through the clinic. In this way, the communication problem could be solved. Or service providers can hire staff from multicultural/diverse communities who can better serve the need of the patient” [FGD # 10, participant # 6].*

Overall, the use of a translator would mitigate language barriers, significantly improving the doctor-patient relationship and allowing for enhanced patient satisfaction. One of the seemingly simplest suggestions to remove language barriers was to hire diverse multilingual professionals from various ethnocultural groups.

*“Culture plays a vital role in healthcare utilization. Medical professionals including doctors here need workplace cultural skill training as Canada is a multicultural country and everyone wants to be respected” [FGD # 9, participant # 4].*

*“You know as a male I cannot talk with a female doctor about my sexual problems or issues on my private part. Nepalese females do not feel comfortable examining their reproductive health with male doctors” [FGD# 5, participant # 2]*.

## Government/Policy Level Approaches

The importance of government-level interventions was widely discussed in the FGDs. The specific levels of government (ie, provincial/federal) were not discussed by the participants. They recommended actions that need to be taken by some level of government that have the responsibility and capacity. All of the FGD participants mentioned that the government (provincial/federal, whichever is appropriate) must create policies to enhance diversity and inclusion in Canadian society. A primary example of how to do this is mandatory cultural competency training for all staff in healthcare service centers, which likely falls under the responsibility of the provincial government.

*“Culture plays a vast role in healthcare use. Medical professionals including doctors here need cultural competency training while they are doing medical training during medical school. Canada is a multicultural country, where everyone from different cultures likes to respect their culture and heritage. So, it would be nice if people are trained in this way from their professional schooling” [FGD# 9, participant # 4].*

Likewise, our participants discussed dental and vision care is expensive, so they suggested that it would help if that could be covered by the government similar to other components of primary care. For this solution, a collective effort by federal and provincial governments will be needed.

*“Dental and vision care are very expensive here. It seems that these sectors are not closely monitored by the government. They are charging differently. So, this sector should be closely monitored by the government and included as a PHC” FGD # 10, participant # 3].*

Finally, participants shared their insight on the untapped skill we have by way of internationally trained medical professionals. Many of these professionals are currently working in jobs that are irrelevant to their medical knowledge. The federal government could provide the necessary training and education required for them to enter the healthcare industry, and ease the workforce shortage, which potentially can improve the quality of healthcare that immigrants receive.

*“There are many internationally graduated health professionals doing different jobs as they found it hard to enter in health industry here. So, the government can make specific refresher courses and curricula to train internationally trained medical professionals. Canada can be benefitted from this type of educational system as this could be a short and effective way to provide refreshing training to experienced professionals. Medical education is expensive around the world” [FGD # 7, participant # 2].*

## Discussion

**Figure 1.**
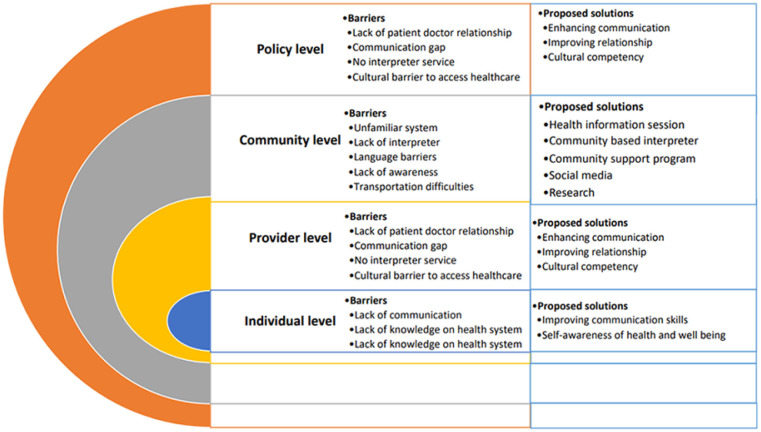
Proposed solutions against the perceived barriers to accessing primary healthcare by Nepalese immigrants.

This research was conducted with immigrant Nepalese community members in Calgary, Alberta, Canada. The research team consisted of community scholars and citizen researchers from the Nepalese community in Calgary. Through focus group discussions with the community members, we sought to explore potential solutions to their experienced barriers to accessing primary care. The potential solutions that FGD participants shared varied in terms of responsibility at different levels, namely individual, community, service provider, and government levels (Figure 1). They believed every sector has its responsibility to assist in mitigating barriers that immigrants face when accessing primary care.

FGD participants acknowledged that the community members can take some initiatives to improve primary care access. For example, participants expressed that people could increase their knowledge of health and navigation of the healthcare system by educating themselves. This refers to the suggestion for improving self-health by being proactive about overcoming barriers to accessing health information by encouraging themselves to use the Internet, reading health brochures, and not solely depending on receiving health information and knowledge from care providers passively. Some strategies on how to maintain health such as attending primary care clinic visits regularly and on time, asking necessary questions to the doctor during appointment time, and following up regularly. A previous study on Bangladeshi-Canadian immigrants also reported similar individual-level initiatives to overcome access to primary care.^21^ However, Canadian Public Health Association reports that a complex web of socio-economic conditions in Canada, which are very prominent for immigrants may affect the individual capacity to be health literate, thereby better access to primary care.^38^

FGD participants also felt that their English language skills and accents prevented them from forming good relationships with medical professionals. This perceived disconnection from care providers may lead to feelings of stress and anxiety around medical visits and often, missed appointments.^39,40^ As an individual-level solution, participants suggested improving English language proficiency by taking classes provided by immigrant societies, practicing language learning with their peers, and getting help from their children. Pandey et al^41^ in their study with immigrants in Saskatchewan, Canada reported that often to overcome language barriers untrained and culturally insensitive interpreters are used, which can be inadequate and sometimes dangerous due to misinterpretation and confidentiality issues. This further reinforces the need for individual improvement of English and assimilation into Canadian culture by individual-level actions as suggested by the participants in this study.

Community-level approaches discussed were health literacy and health education programs, social media use, translation services, participation in community meetings, an orientation program for newcomers, and research programs. A systematic review has identified that a lack of knowledge regarding the existing healthcare system is a barrier to accessing primary care amongst many immigrant communities in Canada.^9^ Our study participants felt that education sessions on the healthcare system of their host country would be useful in increasing health literacy and access among newly landed immigrants. Similarly, a study in a Chinese community in the United States demonstrated that health information workshops positively improved immigrants’ knowledge and awareness of health.^42^ Our participants suggested that Nepalese Canadian residents might have the capacity to organize health information sessions through their community organization (NCSC) in their native language. Doing so in their language of origin is an added benefit as it allows people to have in-depth conversations and have their questions answered in certain terms, building confidence, and reducing the stress of navigating a new system. Health education/information session is the most evident way to improve knowledge and change interrelated health attitudes and behavior among immigrants.^42^

Our FGD participants stressed the importance of using community volunteers as interpreters during healthcare visits for patients with language problems to help express their health concerns clearly to their care providers. Using trained interpreters from their community can express patient health issues to physicians and facilitate communication effectively with maintaining cultural relevance. A study with Portuguese and Spanish immigrants in the US who had limited English language proficiency found that the use of interpreter services changed individual health attitudes and behavior to access healthcare services.^43^ Patients were able to express their concerns in their native language to an interpreter and felt secure that their health concerns were addressed. This resulted in more positive attitudes toward healthcare services and increased service use.^43,44^ This can be a community initiative to support their community members on a voluntary basis, however, the provincial governments can make formal avenues, and as such the interpreters can get compensated for their time improving the sustainability of this approach.

Participants mentioned that they were often not satisfied with the services provided by healthcare professionals largely due to communication and cultural gaps. They expressed that if they were unable to clearly explain their concerns to the doctors, the doctors should have sought adequate support such as trained and culturally competent interpreter services available to assist. Additionally, healthcare professionals from diverse backgrounds should be more involved in providing care to address this gap. Studies noted that doctors can create an environment where patients can freely discuss their health problems without hesitation.^7,45^ For instance, doctors can invite patients to play a more active role in both defining their health concerns and determining suitable treatment plans. In this approach, the doctor’s role is to provide relevant information about available treatment options and their benefits and risks so that the patient can make an informed decision.^45^ It is also important for the service providers to be sensitive to patients’ cultural backgrounds. They suggested that all healthcare professionals, including receptionists, should be well equipped with cultural competence training, making them more responsible and empathetic to a diverse group of patients. One study showed significant positive outcomes among nursing students regarding cultural competence after taking elective cultural competence courses while studying nursing.^46^ These courses led to increased skills in the assessment of cultural variation, communication with people from various cultures, and awareness of personal beliefs, attitudes, and behaviors.^46,47^ Our participants highly valued cultural sensitivity in healthcare. They suggested that as a diverse, multicultural country, Canada should include cultural competency training for all healthcare professionals. A similar study by Kaihlanen et al^48^ reported that nurses felt better prepared to provide effective services to multicultural patients with their diverse healthcare needs after receiving cultural competence training.

Regarding government/policy-level approaches, our FGD participants suggested increasing health resources, including cumulative healthcare facilities and after-hours clinics, employing internationally graduated medical professionals in the healthcare sector, including dental and vision care in primary care, data sharing, and cultural competency training. The participants did not specifically account for the level of governments—provincial or federal during their discussions, however, we acknowledge that some of the recommendations fall under provincial responsibilities while others under federal responsibilities. For example, increasing service hours and facilities fall under the provincial government’s responsibility whereas facilitating the utilization of internationally graduated medical professionals in the healthcare field comes under federal responsibilities (ie, recognition of the skills/credentials/and bridging programs to assimilate them in the healthcare workforce, etc.).^3^ More healthcare facilities and more medical professionals reduce long waiting times too. Additionally, our participants found that dental and vision care is expensive and not strictly regulated and felt that the government should adopt these into primary care, allowing more people to access these necessary services. To increase the number of healthcare workers, participants advised that it is necessary to change medical school entrance policies and provide more funding to encourage visible minority groups who have experienced an understanding of cultural diversity. Our participants suggested that electronic record-keeping and sharing may increase patient satisfaction and assist doctors in walk-in clinics to provide better service to the patient. Many participants thought that electronic record systems also reduce wait time and costs. As Burton et al^49^ found, that electronic health records could fundamentally improve healthcare quality and coordination by exchanging health information and continuously updating patient’s clinical data. Therefore, other physicians can access them easily know the patient’s current and previously treated conditions, the medications they are taking, clinical notes, and diagnostic tests and results for better care that assists other physicians to make clinical decisions.

The key strength of the study is the use of a committed collaborative approach to involving Nepalese community members as community scholars and citizen researchers of this study.^18,19^ We also engaged with the social-cultural organizations and community champions during the study which contributed to increased research in the community.^22^ A limitation of this study is the susceptibility of the FGDs to biases. There is a chance of being influenced by more dominant participants during group discussions. Nevertheless, our FGDs were conducted by an experienced facilitator using FGD guidelines, the facilitator was well aware of dominance and shyness biases. Another limitation is that our study population was constituted of Nepalese immigrants who happened to be also highly educated. This might skew the perspectives away from average Nepalese immigrants. However, due to economic migration, we believe most Nepalese immigrants in Calgary are highly educated. So, while the findings from this study might represent the perspective of the Calgary Nepalese community, they might not be transferable to the overall Nepalese communities in Canada or other immigrant communities. Also, in a focus group setting, the participants might have felt pressured to give responses that are not necessarily their true opinion or understanding but are instead socially accepted. We reassured the participants of confidentiality and that their responses will be published and de-identified, however, some participants may still not be fully expressive of their opinion in front of other participants. While using individual interviews might have been useful to overcome this limitation, interviews are more time-consuming and do not allow for group interaction that helps idea generation through a vibrant discussion.

This community-engaged study highlighted a range of possible actions, which the Nepalese community envisioned would be beneficial to mitigate the primary care access issues they face. At an individual level, a person can make effort to improve their language and communication, as well as try to familiarize themselves with the new healthcare environment they are in. Similarly, community organizations can disseminate healthcare-related information and assist new immigrants to address their healthcare needs. Furthermore, service providers can arrange the necessary healthcare needs of immigrants (e.g., interpreters) and train their healthcare workers to accept multiculturalism and diversity and create an immigrant-friendly environment. The government (federal or provincial) can make flexible policies to provide opportunities for internationally graduated medical professionals to enter the Canadian healthcare system, add more facilities and service centers, and adopt dental and vision care in the universal healthcare system by the provinces and territories. It would be beneficial to do more studies on underprivileged immigrants including refugees to better understand their barriers to accessing primary care and involve them in mitigating those barriers.
